# The impact of COVID-19 on self-reported burnout and health and mental health services in Nampula, Mozambique

**DOI:** 10.3389/fpubh.2022.951270

**Published:** 2022-08-17

**Authors:** Paulino Feliciano, Jennifer J. Mootz, Antonio Suleman, Austin Y. Su, Saida Khan, Lidia Gouveia, Palmira Santos, Milton L. Wainberg, Annika C. Sweetland

**Affiliations:** ^1^Department of Psychiatry and Mental Health, Psychiatric Hospital of Nampula, Nampula, Mozambique; ^2^Department of Psychiatry, New York State Psychiatric Institute, Columbia University, New York, NY, United States; ^3^Columbia Vagelos College of Physicians and Surgeons, New York, NY, United States; ^4^Department of Mental Health, Ministry of Health, Maputo, Mozambique

**Keywords:** Mozambique, COVID-19, mental health, healthcare workers, stigma, burnout, mental health services

## Abstract

**Background:**

The purpose of this study was to examine the impact of the COVID-19 pandemic on self-reported burnout of health workers, quality of care, and perceptions of COVID-19-related stigma in Mozambique.

**Method:**

We conducted a cross-sectional quantitative assessment of 170 frontline health workers in Nampula District, Mozambique, including 149 (87.6%) primary care providers and 21 (12.4%) mental health specialists.

**Results:**

Of the 170 frontline workers, only 10.1% of frontline workers were experiencing more professional burnout, whereas 33.3% felt it had lessened. The perceived impact on quality of care also varied, without any significant differences by sex, education level, or mental health training background. Compared to the beginning of the COVID-19 pandemic in March 2020, 42.3 and 38.1% providers felt that their ability to provide mental health and general health care, respectively, had worsened, compared to 57.7 and 61.9% who believed that either there was no change, or that the work had become easier. Likewise, 26.8% of providers felt that their ability to meet patients' needs had gotten more difficult, whereas 43.4% reported no change and 29.8% reported that it was easier. Twenty-two percent of providers reported an overall increase in caseloads since before the pandemic whereas the majority (67.9%) reported a decrease. Providers believed that 57.1% of people in general were afraid of people with COVID-19, 27.5% were afraid of a person recovered from COVID-19, and 39.9% were afraid of a person with family members with COVID-19. The perceived stigma about healthcare professionals was also low; only 27.4% believed that people in general were afraid of healthcare providers who deliver care to people with COVID-19.

**Conclusion:**

In contrast with other global studies, many healthcare workers in our sample reported a reduction in burnout, which may be associated with the lower overall caseloads seen during this period. Similarly, the quality of care was minimally impacted. More research is needed to determine whether the experience in Mozambique is unique, or similarly observed in other low-resource settings.

## Introduction

The COVID-19 pandemic has had a profound impact on the mental health of directly and indirectly affected individuals. A recent meta-analysis of five countries found the prevalence of symptoms of anxiety, depression, and Post-Traumatic Stress Disorder (PTSD) to be 32.6, 27.6, and 16.7%, respectively (1). In subgroup analyses between confirmed and suspected COVID-19 cases, rates were even higher, at 63.9% for anxiety symptoms and 55.4% for depressive symptoms (1). Frontline healthcare workers are highly vulnerable to the mental health impacts of the pandemic. Widespread pandemic-related burnout has been reported in countries such as the United States (2), Spain (3), Italy (4), China (5), and many others, in both inpatient and outpatient settings (3). In another systematic review and meta-analysis of 38 studies from 19 countries, one-third to one-half of all healthcare professionals had evidence of mental health problems. The combined prevalence of PTSD, anxiety, depression, and distress was 49, 40, 37, and 37%, respectively (6). PTSD may be more prevalent among health workers due to increased stress (7). Factors that mediate pandemic-related burnout in healthcare workers include resilience (8), workplace social support (9), and negative financial and economic impacts (10).

In Mozambique, the first case of COVID-19 was detected in March 2020. The first wave of the pandemic occurred between September and November 2020 with 7,983 confirmed cases and a fatality rate of 1% (11). The second wave was from January to March 2021, during which time an additional 67,197 cases were detected and the fatality rate was 1.1% (11). The timing of waves in Mozambique coincides with the timing of waves in other African countries: 73% of African nations similarly experienced a second, more severe COVID wave in late 2020 through early 2021, with a peak mean daily caseload of 23,790 compared to 18,273 in the first wave (12). It is likely, but not known, that there was an overrepresentation of infected healthcare workers in both waves.

This study had three objectives, which were to assess: (1) the impact of COVID-19 on health and mental health care delivery, (2) levels of burnout among frontline health workers, and (3) the stigma related to COVID-19. The study was carried out in February 2021, at the peak of the second wave before vaccines were available in Mozambique.

## Methods

We received ethical approval for this study from Institutional Review Boards at New York State Psychiatric Institute in the US and Mozambique Institute for Health Education and Research in Mozambique.

### Sample

This study was carried out in Nampula Province, in northern Mozambique in the context of an initiative in which all healthcare workers from a random selection of 8 districts had recently been trained to screen for and deliver several evidence-based mental health interventions (13). One hundred and seventy frontline health professionals were invited to participate in this study and all (100%) agreed to participate; professional background and type of recent mental health training is available for 167 (98.2%). This includes 152 (91.0%) primary care providers (physicians and nurse technicians) and 15 (8.9%) psychiatric technicians, a mid-level mental health specialist professional category that is unique to Mozambique, which involves 30 months of technical academic training to provide mental health services, including prescribing psychotropic medications (14). 4 months prior to the survey, all 152 primary care providers in this sample had received several mental health trainings as part of a research study (13); 77 (45.3%) had received partial mental health training (screening for mental disorders and prescription of psychotropic medications) and 75 (44.1%) were additionally trained to deliver three evidence-based counseling interventions for common disorders, substance use disorders, and suicide risk (comprehensive mental health training). Of the 170 providers, 83 were female (48.5%), 86 were male (50.6%), and one declined to respond. The mean age was 31 years (range 21–65, s.d. 8).

### Data collection

A 6-h training was first provided to the seven research assistants to facilitate data collection via REDCap. The research assistants called providers across eight districts (Erati, Nacaroa, Lipo, Rapale, Mecount, Ribaue, Angoche, Larde) of Nampula Province and inquired about their interest in completing the survey. All primary care providers and psychiatric technicians had received tablets when they completed training for delivery of mental health interventions. For those interested, the research assistants explained the process of completing the survey using their tablet. The research assistants were available to answer any technical questions that arose regarding accessing the survey via the tablet.

### Survey

The survey consisted of 12 items measuring three constructs: (1) the impact of COVID on health and the provision of mental health care; (2) professional burnout; and (3) stigma related to COVID. The first five items asked providers to rate on a 3-point Likert scale the degree of change since the beginning of the pandemic (March 2020) in terms of their ability to provide health and mental health services, quality of care provided, perceived ability to meet the needs of patients and the mental health status of patients. Response options included 1 (worst), 2 (no change), or 3 (best). A sixth item asked them to describe any changes in their patient caseload since March 2020 on a 5 point scale (significantly decreased to significantly increased). Two questions assessed professional burnout: the first asked professionals to rate their level of burnout in general, and the second asked the degree to which their level of burnout had changed over time. Finally, four stigma-related items assessed providers' opinions about the degree to which they thought people in general were afraid of people with COVID-19, people who had previously had COVID-19, family members of people with COVID-19, and/or health workers who see COVID-19 patients based on a 3-point scale (false, a little true, very true).

### Data analysis

We used SPSS 26.0 to summarize and report on demographic characteristics and frequencies in item results. We analyzed the relationship between demographic data (sex, education level, and mental health training) and dependent variable results using the chi-square test for independence. Mental health training was divided into three groups for analysis: partial mental health training, comprehensive mental health training, and psychiatric technicians (mental health specialist). Chi-square values at *p* < 0.05 indicate statistical significance. There were no missing observations for the survey responses about COVID-19.

## Results

### Impact of COVID-19 on healthcare delivery

Most providers indicated that their number of cases had declined since before the pandemic, with just 22% reporting an overall increase. The perceived impact on the quality of mental healthcare delivered varied across the sample, with no significant differences by sex x2 (6, *N* = 170) = 5.480, *p* = 0.484, education level x2 (9, *N* = 170) = 6.817, *p* = 0.656, or mental health training background x3 (6, *N* = 167) = 7.850, *p* =. 0249. When compared to the quality of care provided before March 2020, 42.3 and 38.1% of providers felt that their ability to provide mental health and general health care, respectively, had worsened. Many providers expressed that there was no change in mental health care provision (29.8%) and health care (37.5%) or noticed that their work was becoming easier (28.0 and 24.4%, respectively). Assessments of the patients' mental health status were evenly distributed among the response options: 35.7% thought the patients' mental health had worsened, 28.6% thought there was no change, and 32.1% thought that patients' mental health had improved. Most providers felt that there were no changes in their ability to meet patients' needs (43.4%) or that meeting patients' needs had become easier since March 2020 (29.8%). Fewer providers (26.8%) found that meeting patients' needs had become more difficult.

### Impact of COVID-19 on professional burnout

Of the 170 frontline workers, only 10.1% felt that their burnout at work had worsened. A little more than half (53%) felt that there was no change and a third (33.3%) felt that burnout had improved. There were no significant differences in feelings of burnout according to sex x2 (8, *N* = 170) = 5.032, *p* = 0.754, education level x2 (9, *N* = 170) = 9.479, *p* = 0.394, or mental health training background x3 (8, *N* = 167) = 13.140, *p* = 0.107.

### Stigma related to COVID-19

Regarding the stigma related to COVID-19, providers believed that people were more likely to be afraid of a person who had COVID-19 (57.1%) or has a family member with COVID-19 (39.9 %). Providers indicated that fewer people were afraid of someone who had recovered from COVID-19 (27.5%) or healthcare professionals caring for patients with COVID-19 (27.4%). Providers noted that people may have some stigma toward individuals with family members that have COVID (40.1%).

## Discussion

In this study, we found that, among primary care providers and psychiatric technicians in Nampula, Mozambique, most did not perceive any decrease in their ability to provide mental or primary healthcare or their ability to fill their patients' needs due to the pandemic. That said, they also noted a decrease in the overall number individuals seeking primary healthcare services. In addition, most providers reported unchanged or decreased levels of burnout as compared to before the pandemic.

These findings stand in stark contrast to the negative mental health impact of the pandemic on health-care workers seen elsewhere in the world, including other African nations. A meta-analysis of 27 studies analyzing mental health symptoms in African healthcare providers during the pandemic found an overall prevalence of 45% for depression 37% for anxiety, and 28% for insomnia (15), which are comparable to the rates of pandemic burnout symptoms found in multi-continental studies (6). Factors that mediated burnout in these settings have included poor work environments, interpersonal and professional conflicts, emotional distress, and low social support (16). However, authors have noted a high degree of variability between individual studies, as well as a significantly lower rate of burnout symptoms in Sub-Saharan Africa when compared to North Africa (16), suggesting heterogeneity of burnout symptoms across the continent. Even with these differences and within this context, our findings are unusual in that most of our surveyed providers reported no change or even improvement in burnout symptoms.

In addition, COVID-19 incidence and mortality rates are generally lower in African countries, including Mozambique, which has been theorized to be due to lower mean age and lower average life expectancy paradoxically leading to a younger, more COVID-resilient population (17). However, these rates may also be underreported. A recent study by the COVID-19 Excess Mortality Collaborators estimated the true death toll of the pandemic as up to three times higher than the official toll, and noted that most African countries, including Mozambique, had a high degree of discordance between reported COVID-19 mortality and excess mortality compared to pre-pandemic levels (18). Combined with the limited COVID-19 testing capacity of many resource-poor African nations (19), these data suggests that the true impact of COVID-19 in Africa remains to be seen.

The decrease in patient volume may also be explained by increased barriers to healthcare. Even before the pandemic, healthcare in Nampula was relatively difficult to access due to distance to clinics and poor road networks (20). In addition, many studies reported that the COVID-19 pandemic caused a reduction in health service use, including hospitalizations and clinic visits, across the African continent. Likely mediators have been quarantine and movement restriction policies widely implemented to prevent spread of infection (19). In Mozambique, all but urgent care healthcare services were temporarily suspended across the country to minimize the spread of COVID-19. Moreover, all patients were required to wear masks to receive any healthcare and, although masks were widely available and mandated throughout the country, any individuals who were not able to purchase a mask may not have been able to seek and receive healthcare services.

Another potential factor affecting our findings is COVID-19 stigma, which our providers noted was prevalent within their patient population and in the community in general. COVID-19 stigma is very common in rural areas of Africa due to limited access to mainstream media and widespread health illiteracy resulting in the spread of COVID-19 misinformation (21). As such, it is highly likely that, across Nampula Province, fear of contracting COVID-19 may have made people less likely to seek care. Paradoxically, these factors could have led to increased time and resources per patient, more favorable working conditions, and less pandemic burnout among providers as a result.

There may be alternative explanations of our findings unrelated to decreases in patient volume. First, malaria, tuberculosis, and HIV are endemic to the area, and regions of Mozambique including Nampula had recently seen polio, cholera, and measles outbreaks in the year prior to the pandemic (22). Medical personnel in Mozambique have more familiarity with infectious disease outbreaks and could therefore be less concerned with COVID-19 given the relatively low mortality rate of COVID-19 compared to other conditions such as tuberculosis and HIV.

Another potential protective factor was a recent policy change that reduced the number of hours providers worked; healthcare providers in Nampula were beneficiaries of this policy without a concomitant decrease in pay. This policy could have reduced financial or work-related stressors associated with burnout (16). Finally, it is important to note that our sample consisted of primary care providers who had recently received several trainings in mental health as part of an ongoing study. Positive outcomes of this training, including normalization of mental health issues and/or direct emotional benefit for healthcare workers, may have also had some protective effect against burnout.

This study had several limitations. First, despite reassurances that all responses were confidential, it is possible that some providers modified their responses due to social desirability. Second, there is some uncertainty as to whether providers in Nampula conceptualized “burnout” in the same way that providers in Western nations or higher-income nations might. The idea of burnout was developed in the United States and Europe and has been criticized as ethnocentric due to its framing around job and profession, concepts that may have different cultural contexts in non-Western communities (23). Despite these criticisms, cross-cultural studies of burnout are minimal. Indeed, the Maslach Burnout Inventory, the leading metric for measuring burnout symptoms, has not yet been validated in African populations (16), although it is used widely there. More research focused on cross-cultural interpretations of burnout is needed to maximize the efficacy of mental health research in non-Western nations. Finally, there was a problem in the translation of one question which led to ambiguity in interpreting results. Instead of saying “are people afraid of a person that *has* COVID-19?” the question was worded in the past tense: “are people afraid of a person that *had* COVID-19?” This was nearly identical in meaning to the subsequent question which was “are people afraid of a person that recovered from COVID-19?” Despite the similarities, the proportion of respondents that said yes to the former question (57.1%) was higher than the responses to the latter question (27.5%), suggesting that they interpreted the question in the intended way (current vs. past infection).

In sum, in this typically overburdened and under-resourced setting, COVID-19 cases did not overwhelm the health system, but rather led to a reduction in the number of people seeking medical care, contributing to more favorable working conditions. The improved working conditions may have mitigated the impact of the burnout pandemic among frontline workers, as well as their ability to deliver quality care. More research is needed to determine whether the experience in Mozambique is unique, or similarly observed in other low-resource settings.

## Data availability statement

The raw data supporting the conclusions of this article will be made available by the authors, without undue reservation.

## Ethics statement

The studies involving human participants were viewed and approved by the IRBs of New York State Psychiatric Institute and Mozambique Institute for Health Education and Research. The patients/participants provided their written informed consent to participate in this study.

## Author contributions

PF, JM, AS, ACS, and MW: conceptualization, data collection, data analysis, and writing. AS, SK, LG, and PS: data analysis and writing. All authors contributed to the article and approved the final version.

## Funding

This research was supported by a Fogarty International Center (FIC) and National Institute of Mental Health (NIMH) grant D43 TW009675, PALOP Mental Health Implementation Research Training (Principal Investigators: MW, Emilia Noormahomed, and Maria A. Oquendo), NIMH grant U19 MH113203, PRIDE sSA—Partnerships in Research to Implement and Disseminate Sustainable and Scalable EBPs (Evidence Based Practices) in Sub-Saharan Africa (Principal Investigators: MW and Maria A. Oquendo), and NIMH Mentored Patient-Oriented Research Career Development Award grant K23MH122661-02, Treating Common Mental Disorders in Women in Mozambique by Addressing Intimate Partner Violence in Couples (Principal Investigator: JM).

## Conflict of interest

The authors declare that the research was conducted in the absence of any commercial or financial relationships that could be construed as a potential conflict of interest.

## Publisher's note

All claims expressed in this article are solely those of the authors and do not necessarily represent those of their affiliated organizations, or those of the publisher, the editors and the reviewers. Any product that may be evaluated in this article, or claim that may be made by its manufacturer, is not guaranteed or endorsed by the publisher.
